# Radiomic nomogram for discriminating parotid pleomorphic adenoma from parotid adenolymphoma based on grayscale ultrasonography

**DOI:** 10.3389/fonc.2023.1268789

**Published:** 2024-01-11

**Authors:** Yi Mao, LiPing Jiang, Jing-Ling Wang, Fang-Qun Chen, Wie-Ping Zhang, Zhi-Xing Liu, Chen Li

**Affiliations:** ^1^Department of Ultrasound, The First Affiliated Hospital, Jiangxi Medical College, Nanchang University, Nanchang, Jiangxi, China; ^2^Department of Ultrasound, GanJiang New District Peoples Hospital, Nanchang, Jiangxi, China

**Keywords:** ultrasonography, radiomics, parotid tumor, nomogram, wavelet transformation

## Abstract

**Objectives:**

To differentiate parotid pleomorphic adenoma (PA) from adenolymphoma (AL) using radiomics of grayscale ultrasonography in combination with clinical features.

**Methods:**

This retrospective study aimed to analyze the clinical and radiographic characteristics of 162 cases from December 2019 to March 2023. The study population consisted of a training cohort of 113 patients and a validation cohort of 49 patients. Grayscale ultrasonography was processed using ITP-Snap software and Python to delineate regions of interest (ROIs) and extract radiomic features. Univariate analysis, Spearman’s correlation, greedy recursive elimination strategy, and least absolute shrinkage and selection operator (LASSO) correlation were employed to select relevant radiographic features. Subsequently, eight machine learning methods (LR, SVM, KNN, RandomForest, ExtraTrees, XGBoost, LightGBM, and MLP) were employed to build a quantitative radiomic model using the selected features. A radiomic nomogram was developed through the utilization of multivariate logistic regression analysis, integrating both clinical and radiomic data. The accuracy of the nomogram was assessed using receiver operating characteristic (ROC) curve analysis, calibration, decision curve analysis (DCA), and the Hosmer–Lemeshow test.

**Results:**

To differentiate PA from AL, the radiomic model using SVM showed optimal discriminatory ability (accuracy = 0.929 and 0.857, sensitivity = 0.946 and 0.800, specificity = 0.921 and 0.897, positive predictive value = 0.854 and 0.842, and negative predictive value = 0.972 and 0.867 in the training and validation cohorts, respectively). A nomogram incorporating rad-Signature and clinical features achieved an area under the ROC curve (AUC) of 0.983 (95% confidence interval [CI]: 0.965–1) and 0.910 (95% CI: 0.830–0.990) in the training and validation cohorts, respectively. Decision curve analysis showed that the nomogram and radiomic model outperformed the clinical-factor model in terms of clinical usefulness.

**Conclusion:**

A nomogram based on grayscale ultrasonic radiomics and clinical features served as a non-invasive tool capable of differentiating PA and AL.

## Introduction

Parotid gland tumors are the most common type of salivary gland tumors, with approximately 80%–85% of them being benign. The primary types of these tumors are pleomorphic adenoma (PA) and adenolymphoma (AL) (1), and both of them share similar characteristics, such as slow growth, painlessness, and well-defined borders. However, differentiations between PA and AL are crucial for clinical diagnosis and treatment. On radiological examinations, AL shows more heterogeneous density and signal compared to PA, often accompanied by multiple small cystic changes and increased blood flow (2). PA is more likely to occur in the deep regions of the parotid gland, typically presenting as lobulated, with a higher risk of malignant transformation and recurrence (3). Therefore, PA usually requires a tumor and superficial parotidectomy, along with facial nerve dissection. AL, however, typically only requires partial parotidectomy. However, in PA cases, tumor cells can be detected at the resection margins in 41.9% of cases (4). This could be one of the reasons why PA is more prone to relapse. To distinguish between the two types of parotid gland tumors at an early stage, a fine-needle aspiration biopsy (FNAB) is commonly used as an auxiliary diagnostic tool (5). It has high accuracy in the diagnosis of both benign and malignant tumors. However, FNAB is an invasive procedure and carries the risk of needle-track seeding and facial nerve palsy (6).

Ultrasonic examinations can reflect differences in signal scattering and speckling patterns, which correlate with variations in parotid gland morphology and increasing tissue stiffness (2). Compared to FNAB, grayscale ultrasonic examination is a non-invasive, cost-effective, and user-friendly imaging technique. However, differentiating between PA and AL using grayscale ultrasonic examination can be challenging for sonographers. Some morphological features, long-to-short diameter ratio (L/S) ratio, and ultrasonographic shear wave elastography have limited utility in distinguishing between the two types (7, 8). Therefore, visible differences discernible by the naked eye do not significantly improve the diagnostic accuracy of medical imaging.

Radiomics is a rapidly growing discipline that utilizes machine learning to extract quantitative information from medical images like CT, MR, US, and predict outcomes in cancer research (9–11). For head and neck tumors, radiomic features from T2-weighted MR imaging (T2WI) and contrast-enhanced T1-weighted MR imaging (CE-T1WI) can predict cancer staging pre-operatively (12). Additionally, radiomic features from CT and PET scans can accurately determine if oropharyngeal squamous cell carcinoma is infected with the HPV (P16) virus (13). Radiomics has also shown success in assessing early treatment effects (14) and radiotherapy complications in nasopharyngeal cancer (15). In summary, radiomic analysis of various medical imaging modalities holds potential for improving diagnosis, prognosis, and personalized treatment of head and neck cancers.

Wavelet transformation is created *via* dilatation and translation of the mother wavelet (16). These modifications provide a spatial/frequency representation of the signal, indicating that the wavelet coefficients act as a projection of the original signal onto a multi-resolution subspace. The high-pass filter also draws attention to the grayscale changes in the image, improving the presentation of image details and texture information. The low-pass filter, however, blurs the differences in grayscale, obscuring the finer details of the image and emphasizing its main characteristics (17). The radiomic model’s texture features can be separated further. Studies have shown that, compared to the original radiomics, wavelet-transformed radiomics perform better in assessing COVID-19 lung lesions (18).

The purpose of our study is to investigate whether radiology based on grayscale ultrasonography can distinguish PA and AL and whether the nomogram combined with clinical and radiological features can facilitate and accurately help to distinguish these two benign tumors.

## Materials and methods

### Ethics statement

This study adhered to the principles outlined in the Declaration of Helsinki and received approval from the local ethics review board. Written, informed consent was obtained from all participants.

### Selection of participants

We retrospectively analyzed patients with parotid tumors undergoing grayscale ultrasonic examination at the local hospital from December 2019 to March 2023. The inclusion criteria were as follows: 1) preoperative two-dimensional ultrasonography confirmed the presence of a parotid tumor. 2) A postoperative histopathological examination confirmed the diagnosis of PA and AL. 3) There was no history of fine-needle aspiration (FNA), radiotherapy, or other treatments. 4) Complete clinical and data records were available. 5) A preoperative ultrasound examination was performed within 1 week. The exclusion criteria were as follows: 1) the maximum diameter of the tumor was less than 1 cm; 2) the images were not clear, with incomplete visualization of the tumor and significant artifacts; 3) the concurrent presence of other organ tumors.

### Ultrasonography procedures

The bilateral parotid glands were scanned using high-end ultrasound diagnostic equipment such as Siemens ACUson Sequoia, GE LOGIQ E11, and Philips EPIQ 7. A high-frequency linear array probe was used for the examination. The maximum diameter of the parotid gland masses was saved in the machine’s memory in a cross-sectional view and exported in DICOM format for subsequent analysis.

### Image segmentation

All ultrasound images were imported into the ITK-SNAP (http://www.itksnap.org) software. Two ultrasound physicians with 6 years of experience in the field delineated the tumor margins by carefully outlining them and selecting the maximum section of the tumor to delineate a region of interest (ROI). The delineation was subsequently reviewed and approved by a senior physician. In case of any disagreements, a group discussion was held to reach a consensus.

### Feature extraction

The images and ROIs extracted from the ITK-SNAP software were imported into Python (version 3.11) for further analysis. Handcrafted features were extracted using an in-house feature analysis program implemented in Pyradiomics (https://pyradiomics.readthedocs.io). These features can be categorized into three groups: I) geometry, II) intensity, and III) texture. There were 14 geometry features, 306 intensity features, and 1,241 texture features comprised of the Gray Level Co-Occurrence Matrix (GLCM), Gray Level Dependence Matrix (GLDM), Gray Level Run Length Matrix (GLRLM), Gray Level Size Zone Matrix (GLSZM), and Neighborhood Gray Tone Difference Matrix (NGTDM).

The ROIs were delineated by two sonographers, and the interobserver agreement was evaluated using the interclass correlation coefficient (ICC) analysis. ICC values higher than 0.75 were considered to have good consistency and were selected for further analysis. Patients were randomly divided into two cohorts with a ratio of 7:3 for training and validation purposes, respectively.

### Feature selection

After applying z-score normalization, the t-test and Mann–Whitney U test were performed on all radiomic features. Only features with a p-value <0.05 were retained. For features exhibiting high repeatability, Spearman’s rank correlation coefficient was used to assess the correlation between features. If the correlation coefficient between any two features exceeded 0.9, only one of them was retained.

To identify the optimal feature subset, the least absolute shrinkage and selection operator (LASSO) (19) algorithm was employed. LASSO shrinks all regression coefficients toward zero and sets the coefficients of irrelevant features to exactly zero. A 10-fold cross-validation with minimum criteria was used to determine the optimal lambda (λ) value, which yielded the lowest cross-validation error.

### Model construction and validation

#### Radiomic and clinical models

After performing LASSO feature screening, the final selected features were input into machine learning models such as LR, SVM, RandomForest, and XGBoost. The coefficients of the features were used to calculate a radiomic quality signature (rad-Signature). Clinical features used for building the same machine learning models were selected based on a baseline statistic with a p-value <0.05.

#### Radiomic nomogram

A radiomic nomogram was developed by combining the radiomic signature and clinical features. The diagnostic efficacy of the radiomic nomogram was tested in the validation cohort, and receiver operating characteristic (ROC) curves were plotted to evaluate its diagnostic performance. Calibration curves were used to evaluate the calibration efficiency of the nomogram, and the Hosmer–Lemeshow analytical fit was employed to assess its calibration ability. Additionally, decision curve analysis (DCA) was used to evaluate the clinical utility of the predictive models.

### Statistical analyses

Statistical analysis of the data was performed using SPSS 26.0 and Python 3.11. Continuous variables are presented as mean ± standard deviation, while categorical variables are reported as counts (n). The independent samples t-test was used to analyze clinical data, and the chi-square test was applied for categorical variables. A significance level of p < 0.05 was considered statistically significant.

## Results

### Clinical characteristics

**Figure 1:**
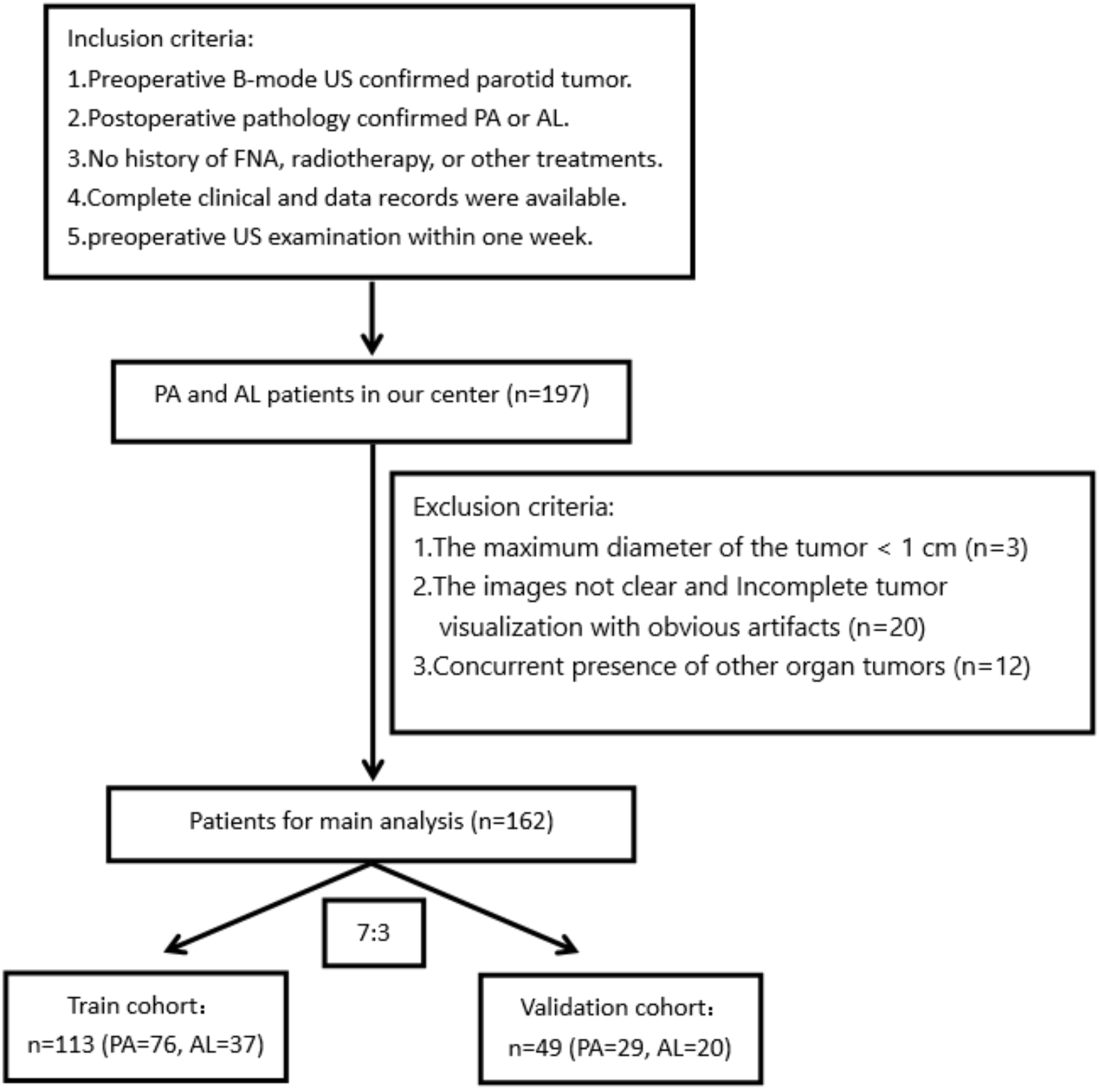
The flowchart of the patient selection process.

The flowchart depicting the process of patient selection is presented in **Figure 1**. **Table 1** displays the clinical and imaging data of the 162 patients included in this study. Out of the total, 105 were confirmed to have PA, and 57 were diagnosed with adenoid cystic carcinoma (AL). The clinical characteristics of all 162 subjects are summarized in **Table 1**. In the PA group, the average age was 43.49 ± 15.67 years, with a male-to-female gender ratio of 0.91:1. Among the AL patients, the average age was 61.50 ± 10.08 years, and the male-to-female gender ratio was 10.5:1.

**Table 1:** Patient’s characteristics at baseline.

Feature name		Train	PA	AL	p-Value	Test	PA	AL	p-Value
Age		49.17 ± 16.74	42.82 ± 15.40	62.22 ± 10.87	<0.001	50.86 ± 16.16	44.28 ± 17.12	60.40 ± 8.10	<0.001
Max D		25.04 ± 9.33	23.30 ± 8.94	28.62 ± 9.21	0.004009086	25.88 ± 8.35	22.38 ± 7.51	30.95 ± 6.88	<0.001
Sex					<0.001				0.001820478
	Male subjects	67 (59.29)	33 (43.42)	34 (91.89)		33 (67.35)	14 (48.28)	19 (95.00)	
	Female subjects	46 (40.71)	43 (56.58)	3 (8.11)		16 (32.65)	15 (51.72)	1 (5.00)	
Smoking					<0.001				<0.001
	No	77 (68.14)	63 (82.89)	14 (37.84)		29 (59.18)	24 (82.76)	5 (25.00)	
	Yes	36 (31.86)	13 (17.11)	23 (62.16)		20 (40.82)	5 (17.24)	15 (75.00)	
Number					<0.001				0.013690557
	Single	93 (82.30)	70 (92.11)	23 (62.16)		39 (79.59)	27 (93.10)	12 (60.00)	
	Multiple	20 (17.70)	6 (7.89)	14 (37.84)		10 (20.41)	2 (6.90)	8 (40.00)	
Position					0.629061941				0.842154851
	Right	62 (54.87)	40 (52.63)	22 (59.46)		29 (59.18)	18 (62.07)	11 (55.00)	
	Left	51 (45.13)	36 (47.37)	15 (40.54)		20 (40.82)	11 (37.93)	9 (45.00)	

PA, parotid pleomorphic adenoma; AL, adenolymphoma.

The 162 subjects were randomly divided into training and validation cohorts in a 7:3 ratio. Therefore, the training cohort comprised 113 cases (76 PA and 37 AL), while the remaining 49 patients (29 PA and 20 AL) were assigned to the validation cohort. There were no significant differences in clinical features between the training and validation cohorts (p-value <0.05).

### Feature selection, model construction, and validation

**Figure 2:**
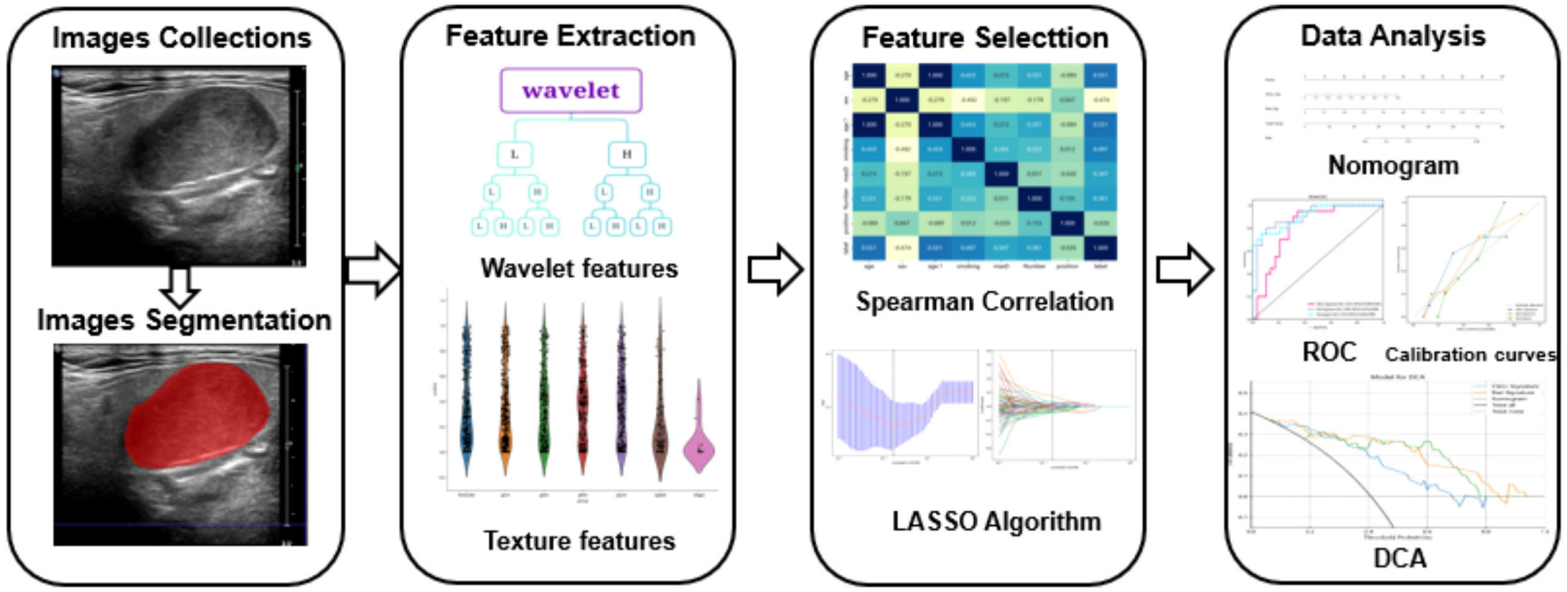
The flowchart detailing the radiomic processing steps employed in this study. The collected images were exported to ITK software for region of interest (ROI) delineation and image segmentation. Ultrasound radiomics were then extracted using Python software. Models were developed based on the clinical features of patients with pleomorphic adenoma (PA) or adenoid cystic carcinoma (AL). The models underwent calibration and validation processes to evaluate their performance.

**Figure 3:**
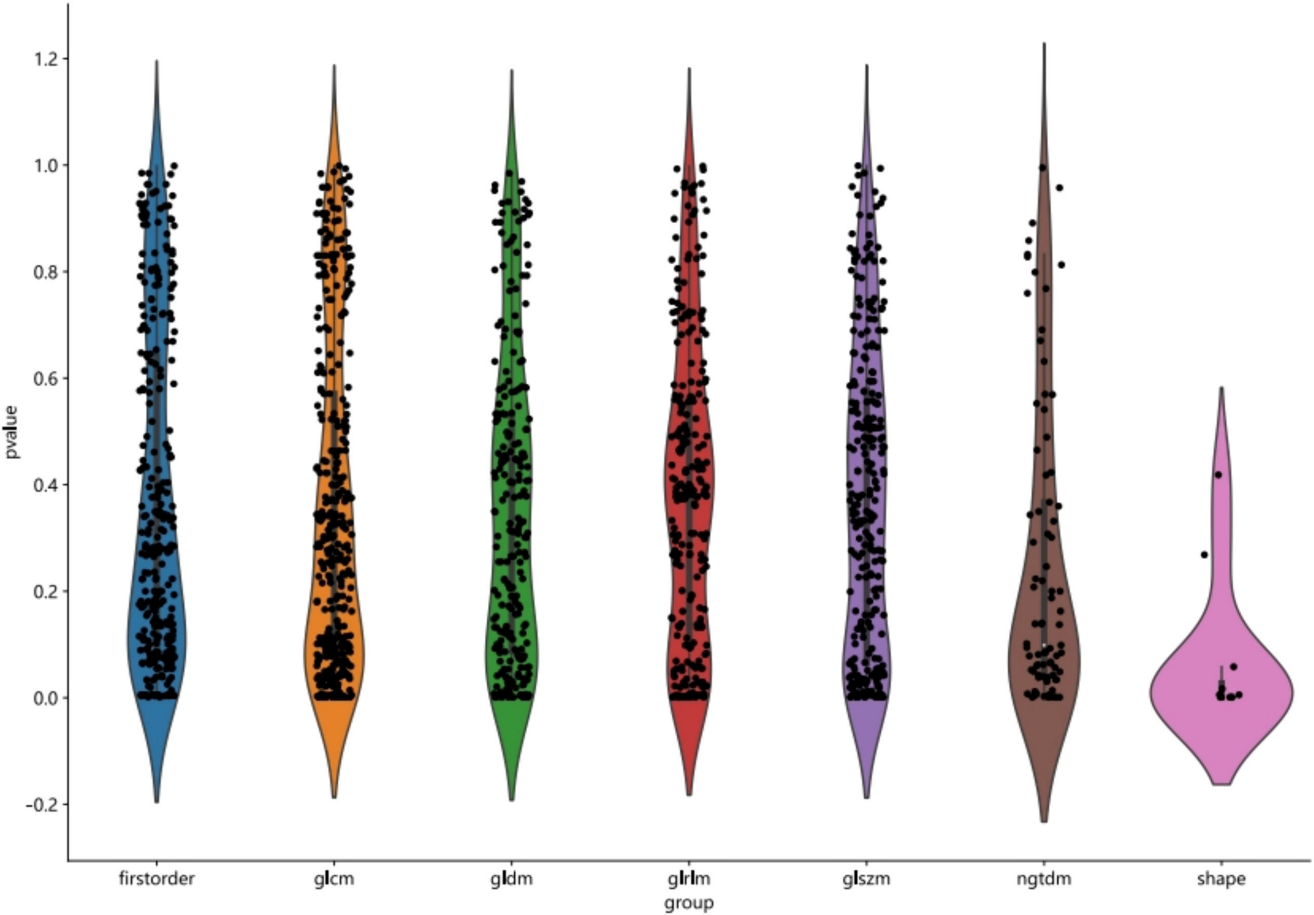
Statistics of radiomic features.

**Figure 4:**
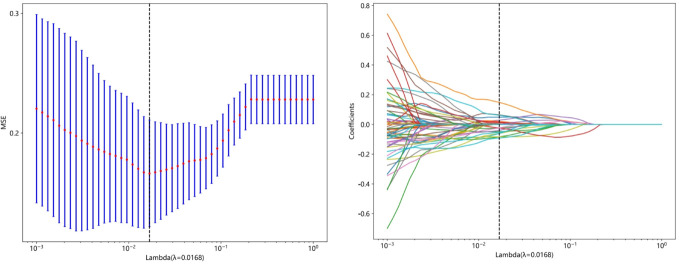
The least absolute shrinkage and selection operator (LASSO) algorithm was employed for feature selection. In the LASSO model, a 10-fold cross-validation approach was utilized to determine the optimal tuning parameter (λ). The minimum criterion was used to select the best values, and vertical lines were drawn to indicate the true selection points. Additionally, a 10-fold cross-validation was performed to identify the selected value in the λ sequence, resulting in 18 features with non-zero coefficients.

**Figure 5:**
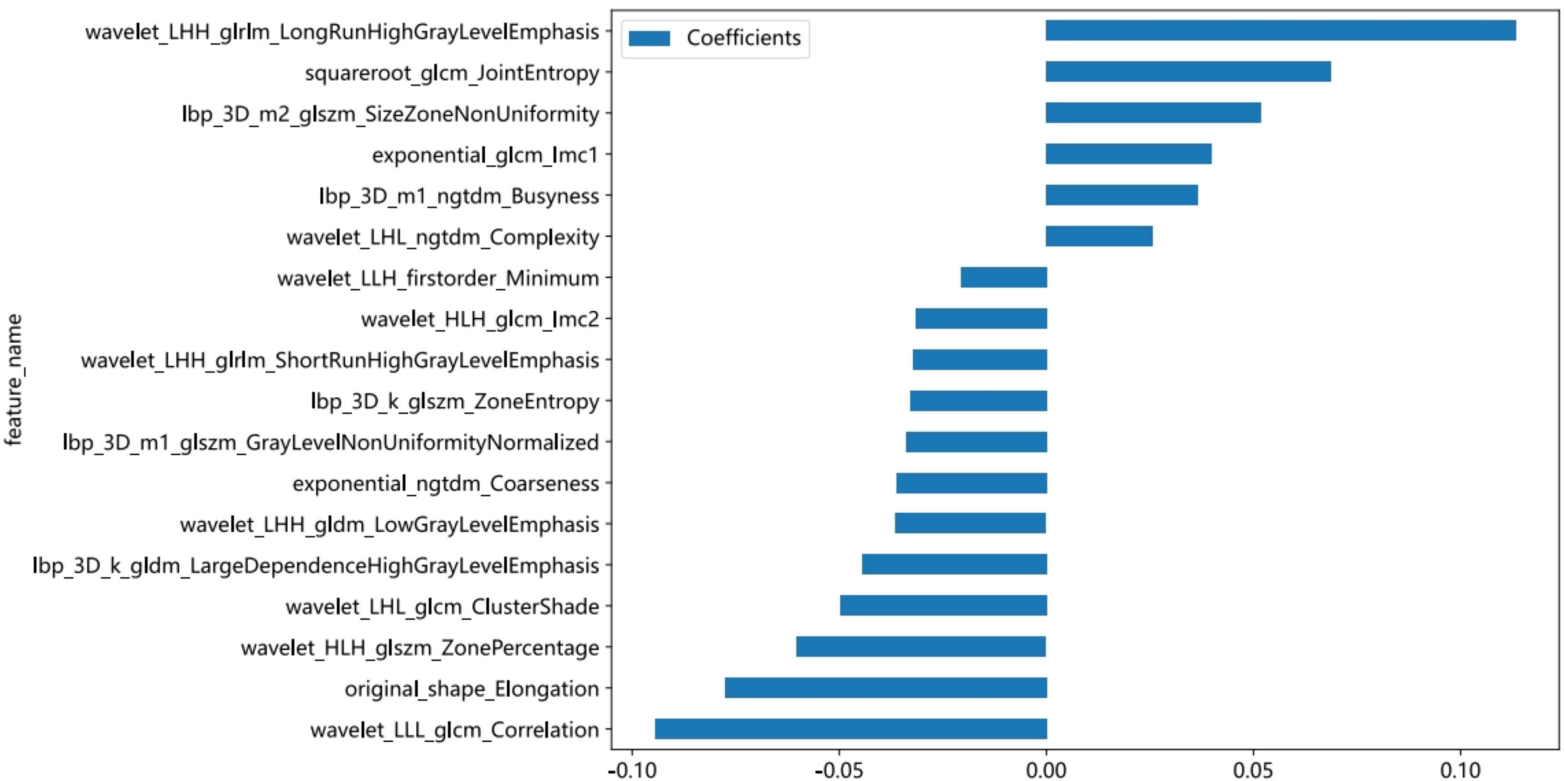
The histogram of the rad-Signature based on the selected features.

The course of processing radiomics is shown in **Figure 2**. From the grayscale ultrasonography for each participant, 1,561 radiomics were extracted; 294 features were selected after univariate analysis and ICC; and70 features were retained after being filtered using Spearman’s correlation (**Figure 3**; Spearman’s correlation of each feature). The radiomic feature selection was performed using LASSO logistic regression, resulting in 18 selected radiomic features. The coefficients and mean standard error (MSE) from the 10-fold validation are presented in **Figure 4**. These features were utilized to construct the radiomic signature. The final formula for calculating rad-Signature and the corresponding coefficients is depicted in **Figure 5**.

### Nomogram performance and validation

**Figure 6:**
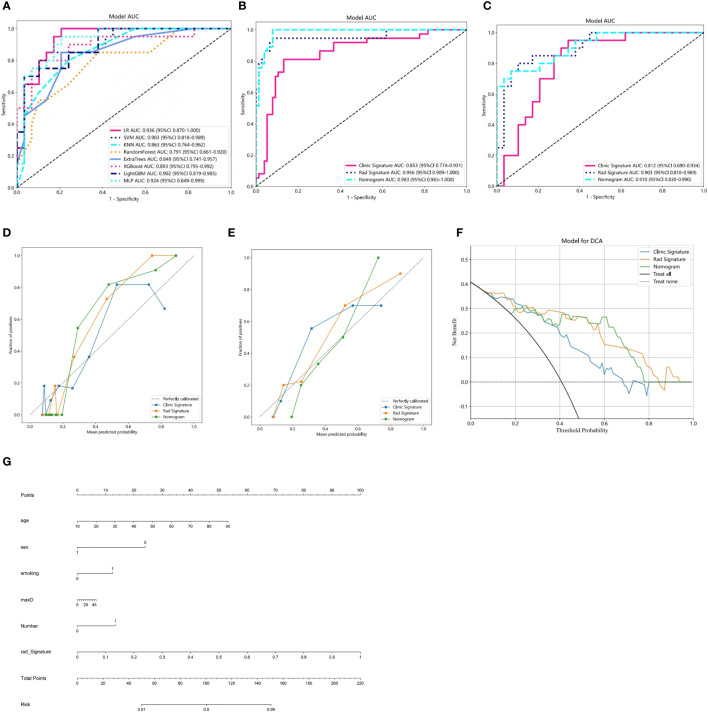
**(A)** Receiver operating characteristic (ROC) curves of the eight classifier models on the validation cohort. **(B, C)** ROC charts of clinical and radiomic models and nomogram performance on the training and validation cohorts. **(D, E)** Calibration curves of clinical and radiomic models and nomogram performance on the training and validation cohorts. **(F)** Decision curve analysis (DCA) of clinical and radiomic models and nomogram performance on the training and validation cohorts. **(G)** Nomogram for clinical features combined with Rad-Signature.

The model constructed using clinical features such as age, maximum diameter, and smoking status showed good predictive performance (accuracy = 0.850 and 0.776, sensitivity = 0.811 and 0.950, specificity = 0.868 and 0.679, positive predictive value = 0.750 and 0.655, and negative predictive value = 0.904 and 0.950 in the training and validation cohorts, respectively). Similarly, the imaging-based radiomic features (**Table 2**), especially the SVM model, demonstrated excellent predictive performance (accuracy = 0.929 and 0.857, sensitivity = 0.946 and 0.800, specificity = 0.921 and 0.897, positive predictive value = 0.854 and 0.842, and negative predictive value = 0.972 and 0.867 in the training and validation cohorts, respectively). Furthermore, incorporating the results of the radiomic model into the clinical model improved the predictive performance of the combined model (accuracy = 0.947 and 0.857, sensitivity = 1 and 0.950, specificity = 0.921 and 0.931, positive predictive value = 0.860 and 0.882, and negative predictive value = 1 and 0.844 in the training and validation cohorts, respectively). The performance comparison of the three models is presented in **Table 3** and **Figure 6** (DeLong test, p < 0.005 for the training and validation cohorts). A nomogram combining clinical features and rad-Signature was developed (**Figure 6**), and its calibration curve demonstrated consistent predictive and observed effects in both the training and validation cohorts.

**Table 2:** Performance contributions of various radiological classifier models in classification.

Model name	Cohort	ACC	AUC	95% CI	SEN	SPE	PPV	NPV	Precision	Recall	F1	Threshold
LR	Train	0.858	0.898	0.838, −0.958	0.730	0.921	0.818	0.875	0.818	0.730	0.771	0.417
	Validation	0.878	0.936	0.870, −1	1	0.793	0.769	1	0.769	1	0.870	0.181
SVM	Train	0.929	0.956	0.909, −1	0.946	0.921	0.854	0.972	0.854	0.946	0.897	0.230
	Validation	0.857	0.903	0.818, −0.989	0.800	0.897	0.842	0.867	0.842	0.800	0.821	0.420
KNN	Train	0.814	0.895	0.840, −0.951	0.784	0.829	0.690	0.887	0.690	0.784	0.734	0.400
	Validation	0.776	0.863	0.764, −0.962	0.800	0.786	0.696	0.846	0.696	0.800	0.744	0.400
RandomForest	Train	1	1	1, −1	1	1	1	1	1	1	1	0.500
	Validation	0.714	0.791	0.661, −0.920	0.850	0.621	0.607	0.857	0.607	0.850	0.708	0.300
ExtraTrees	Train	1	1	1, −1	1	1	1	1	1	1	1	1
	Validation	0.816	0.849	0.741, −0.957	0.850	0.793	0.739	0.885	0.739	0.850	0.791	0.400
XGBoost	Train	1	1	1, −1	1	1	1	1	1	1	1	0.539
	Validation	0.857	0.893	0.795, −0.992	0.750	0.931	0.882	0.844	0.882	0.750	0.811	0.384
LightGBM	Train	0.920	0.977	0.957, −0.998	0.973	0.895	0.818	0.986	0.818	0.973	0.889	0.314
	Validation	0.857	0.902	0.819, −0.985	0.700	0.966	0.933	0.824	0.933	0.700	0.800	0.443
MLP	Train	0.862	0.923	0.877, −0.969	0.804	0.893	0.804	0.893	0.804	0.804	0.804	0.392
	Validation	0.875	0.861	0.716, −1	0.818	0.950	0.818	0.905	0.818	0.818	0.818	0.491

ACC, accuracy; SEN, sensitivity; SPE, specificity; PPV, positive predictive value; NPV, negative predictive value; AUC, area under the receiver operating characteristic curve.

**Table 3:** Performance contributions of three different models in classification.

Cohort	Signature	ACC	AUC	95% CI	SEN	SPE	PPV	NPV	Precision	Recall	F1	Threshold
Train	Clinic	0.850	0.853	0.774, −0.931	0.811	0.868	0.750	0.904	0.750	0.811	0.779	0.370
	Rad	0.929	0.956	0.909, −1	0.946	0.921	0.854	0.972	0.854	0.946	0.897	0.230
	Nomogram	0.947	0.983	0.965, −1	1	0.921	0.860	1	0.860	1	0.925	0.277
Validation	Clinic	0.776	0.812	0.690, −0.934	0.950	0.679	0.655	0.950	0.655	0.950	0.776	0.194
	Rad	0.857	0.903	0.818, −0.989	0.800	0.897	0.842	0.867	0.842	0.800	0.821	0.420
	Nomogram	0.857	0.910	0.830, −0.990	0.750	0.931	0.882	0.844	0.882	0.750	0.811	0.440

ACC, accuracy; SEN, sensitivity; SPE, specificity; PPV, positive predictive value; NPV, negative predictive value; AUC, area under the receiver operating characteristic curve.

To assess the calibration ability of the developed nomogram, the Hosmer–Lemeshow test (20) was employed. The results indicated a good model fit (p-value >0.05), suggesting that the nomogram accurately captured the observed data and that there was no significant difference between the predicted and observed outcomes. The DCA of the nomogram is depicted in **Figure 6F**. Furthermore, the DCA of the nomogram demonstrated a larger area under the curve compared to the clinical model. This indicates that both the nomogram and radiomic model have a greater net benefit in distinguishing between PA and AL.

## Discussion

In this study, we utilized radiomic features extracted from grayscale ultrasonography to assist in the early preoperative differentiation of two benign tumors, PA and AL, with the goal of aiding clinicians in selecting appropriate diagnostic and treatment approaches. Previous studies have demonstrated that there are important differences between PA and AL in terms of clinical features and traditional parameters, including smoking history, age, and the presence of multiple lesions (15). However, the effectiveness of these factors in a comprehensive analysis is inconsistent, with varying areas under the ROC curve (AUC) values ranging from 0.68 to 0.95, leading to significant uncertainty in clinical diagnosis and treatment. Additionally, common ultrasound features such as the L/S and ultrasound grayscale ratio (UGSR) have also shown poor performance (AUC = 0.74) (7). In contrast, the radiomic SVM-based model that we constructed has demonstrated excellent performance in distinguishing between PA from AL, with AUC values of 0.956 in the training cohort and 0.903 in the validation cohort. Additionally, other models in our study have also demonstrated good performance in distinguishing between the two types of tumors, but for the RandomForest, ExtraTrees, and XGBoost models, there are significant differences in AUC between the training and validation cohorts, indicating overfitting of the models (11, 21). However, it is important to note that models with a large number of input parameters or high degrees of freedom may have a tendency to overfit the data by memorizing it. Consequently, when analyzing the features, the model may react to random fluctuations in the data, which is undesirable in accurate feature analysis.

Ultrasound images displaying both PA and AL appear on ultrasound as localized enlargements of the salivary gland with regular morphology and well-defined borders, presenting as well-circumscribed hypoechoic masses. Matsuda (22) indicated that 63.2% of PA cases belonged to the category of no anechoic area homogeneous tumors, while 53.3% of AL cases were classified as multiple and sponge-like anechoic area heterogeneous tumors. However, Jiang (23) and Rong (2) believed that there were no statistically significant differences observed in the sonographic features of boundaries, echo pattern, homogeneity, calcification, and distal acoustic enhancement between PA and AL. We believe that this discrepancy is only related to the number of samples. However, it is undeniable that AL is more susceptible to infection and cystic degeneration, characterized by a loose tissue texture with numerous small cysts that create echo-free areas. In this study, most of the features used for modeling were obtained through wavelet transformation, revealing more layered variation and information content in these feature maps. Among the 18 features used to construct the radiomic model, the most influential feature is wavelet_LHH_glrlm_LongRunHighGrayLevelEmphasis, which describes the texture feature of long and high gray-level runs in the image. A higher value indicates the presence of longer and higher gray-level continuous texture features in the image (24). In our study, the feature value PA > AL can be seen in both the training and validation cohorts (Appendix 1, the average feature values of 18 modeling features in the training and validation sets). We believe that the characteristic of cystic lesions in AL results in a lower value of this feature compared to PA.

Previous studies have shown that radiomic research using CT and MR images performs well in differentiating PA and AL. Zheng (25) gathered 76 instances of PA and 34 cases of AL and built a model based on CT images with an AUC of 0.89 and an accuracy of 83.3%. Song (26) built a T1-2WI model based on MR images with an AUC of 0.90 and an accuracy of 86% after collecting 140 instances of PA and 112 cases of AL. The mutual information (MI) feature model that Fruehwald-Pallamar et al. (27) developed using CE-T1WI pictures had an accuracy of 81.8%. She gathered 13 cases of PA and 11 cases of AL. Similarly, Piludu et al. (28) enrolled 35 parotid PA and 20 AL to construct an SVM model using T2WI and ADC pictures, which was successful with an accuracy of 91.7%. Additionally, according to their studies, AL and PA could possibly be distinguished from one another on T1WI, T2WI, and ADC images by the characteristics of AL’s cystic components.

Our study still established and validated a novel prognostic model using a nomogram-based approach to differentiate between PA and AL. The nomogram, as a predictive statistical model, not only provides a visual display of the relevant indicators influencing the outcomes in multiple regression analysis but also enables a simple graphical representation to predict survival probability, making the prediction simpler and more convenient (29, 30). We combined clinical features and rad-Signature and utilized a nomogram for prediction. The results showed that in both the training and validation cohorts, the AUC was higher than that of the single model. However, in the validation cohort, the specificity was 0.931 while the sensitivity was only 0.750, indicating high accuracy in identifying AL patients. Therefore, this prognostic model has certain clinical applicability. Zheng (25) developed and validated a novel prognostic model using a nomogram-based approach to differentiate between PA and AL. This model incorporated the CT Rad-score and independent clinical factors. The nomogram exhibited excellent discriminative performance, with an AUC of 0.98 in the training cohort and 0.95 in the validation cohort. However, when compared to the CT radiomic model (with an AUC of 0.89 in both the training and validation cohorts), the grayscale ultrasonography-based radiomic model in this study demonstrates higher accuracy and stability.

Nevertheless, our study has several limitations that should be noted. First, due to difficulties in disease epidemiology and obtaining qualified patient images, the sample size was limited, and we did not conduct an independent external validation. Future research should involve a larger dataset for further investigation. Second, our radiomic study only utilized conventional grayscale ultrasonography, which is the most commonly used scanning method. In the future, we plan to incorporate more scanning technologies, such as Sound-Touch Elastography (STE) and contrast-enhanced ultrasound, to construct a multimodal radiomic model to further assist clinical diagnosis and treatment. Third, all images in our study were obtained from a single center. Therefore, we intend to include more types of devices and data centers in future studies to establish a multicenter radiomic model.

## Conclusion

Evaluating the imaging features of grayscale ultrasonography can significantly improve the diagnostic ability of clinical indicators for distinguishing between PA and AL. Based on this, the construction of a nomogram combining radiological features with clinical characteristics is also a highly accurate and non-invasive tool for distinguishing these two benign tumors.

## Data availability statement

The datasets presented in this article are not readily available because the institution requires full protection of patient privacy. Requests to access the datasets should be directed to CL or Z-XL at 1727237899@qq.com; 1214582369@qq.com.

## Ethics statement

Written informed consent was obtained from the individual(s) for the publication of any potentially identifiable images or data included in this article.

## Author contributions

YM: Data curation, Methodology, Software, Supervision, Validation, Visualization, Writing – original draft, Writing – review & editing. LJ: Data curation, Funding acquisition, Investigation, Supervision, Validation, Writing – original draft, Writing – review & editing. J-LW: Writing – original draft. F-QC: Data curation, Investigation, Software, Writing – original draft. W-PZ: Data curation, Investigation, Software, Writing – original draft. Z-XL: Data curation, Funding acquisition, Software, Supervision, Validation, Writing – review & editing. CL: Investigation, Software, Supervision, Writing – review & editing.
