# Supplementary material for: Radiomic nomogram for discriminating parotid pleomorphic adenoma from parotid adenolymphoma based on grayscale ultrasonography
Source: Front Oncol. 2024 Jan 11;13:1268789. doi: 10.3389/fonc.2023.1268789 (PMC10808803; doi:10.3389/fonc.2023.1268789)
Supplement: Supplementary file 1 [file Table_1.docx]

|  | train | |  | validation | |
| --- | --- | --- | --- | --- | --- |
|  | PA | AL |  | PA | AL |
| exponential_glcm_Imc1 | -0.33 | -0.29 |  | -0.34 | -0.28 |
| exponential_ngtdm_Coarseness | 0.0022 | 0.0014 |  | 0.0021 | 0.0010 |
| lbp_3D_k_gldm_LargeDependenceHighGrayLevelEmphasis | 72.33 | 71.27 |  | 71.94 | 71.34 |
| lbp_3D_k_glszm_ZoneEntropy | 3.93 | 4.05 |  | 3.97 | 4.12 |
| lbp_3D_m1_glszm_GrayLevelNonUniformityNormalized | 0.50 | 0.47 |  | 0.48 | 0.41 |
| lbp_3D_m1_ngtdm_Busyness | 613.50 | 1086.05 |  | 860.01 | 1464.59 |
| lbp_3D_m2_glszm_SizeZoneNonUniformity | 74.90 | 110.18 |  | 93.78 | 119.91 |
| original_shape_Elongation | 0.68 | 0.58 |  | 0.72 | 0.58 |
| squareroot_glcm_JointEntropy | 11.24 | 11.44 |  | 11.10 | 11.66 |
| wavelet_HLH_glcm_Imc2 | 0.18 | 0.16 |  | 0.18 | 0.17 |
| wavelet_HLH_glszm_ZonePercentage | 0.01 | 0.01 |  | 0.01 | 0.01 |
| wavelet_LHH_gldm_LowGrayLevelEmphasis | 0.61 | 0.60 |  | 0.61 | 0.61 |
| wavelet_LHH_glrlm_LongRunHighGrayLevelEmphasis | 26.15 | 29.36 |  | 24.80 | 28.93 |
| wavelet_LHH_glrlm_ShortRunHighGrayLevelEmphasis | 1.35 | 1.31 |  | 1.36 | 1.30 |
| wavelet_LHL_glcm_ClusterShade | -663880.58 | -1729800.78 |  | -755680.20 | -1531938.67 |
| wavelet_LHL_ngtdm_Complexity | 8409985.12 | 13142481.45 |  | 10150334.83 | 13582669.30 |
| wavelet_LLH_firstorder_Minimum | -2.39621E-13 | -2.76344E-13 |  | -2.59838E-13 | -2.91411E-13 |
| wavelet_LLL_glcm_Correlation | 0.91 | 0.90 |  | 0.92 | 0.89 |

The values of 18 modeling features in the training and validation sets
